# Supplementary figures and images for: Loss of tumor suppressor WWOX accelerates pancreatic cancer development through promotion of TGFβ/BMP2 signaling
Source: Cell Death Dis. 2022 Dec 27;13(12):1074. doi: 10.1038/s41419-022-05519-9 (PMC9792466; doi:10.1038/s41419-022-05519-9)

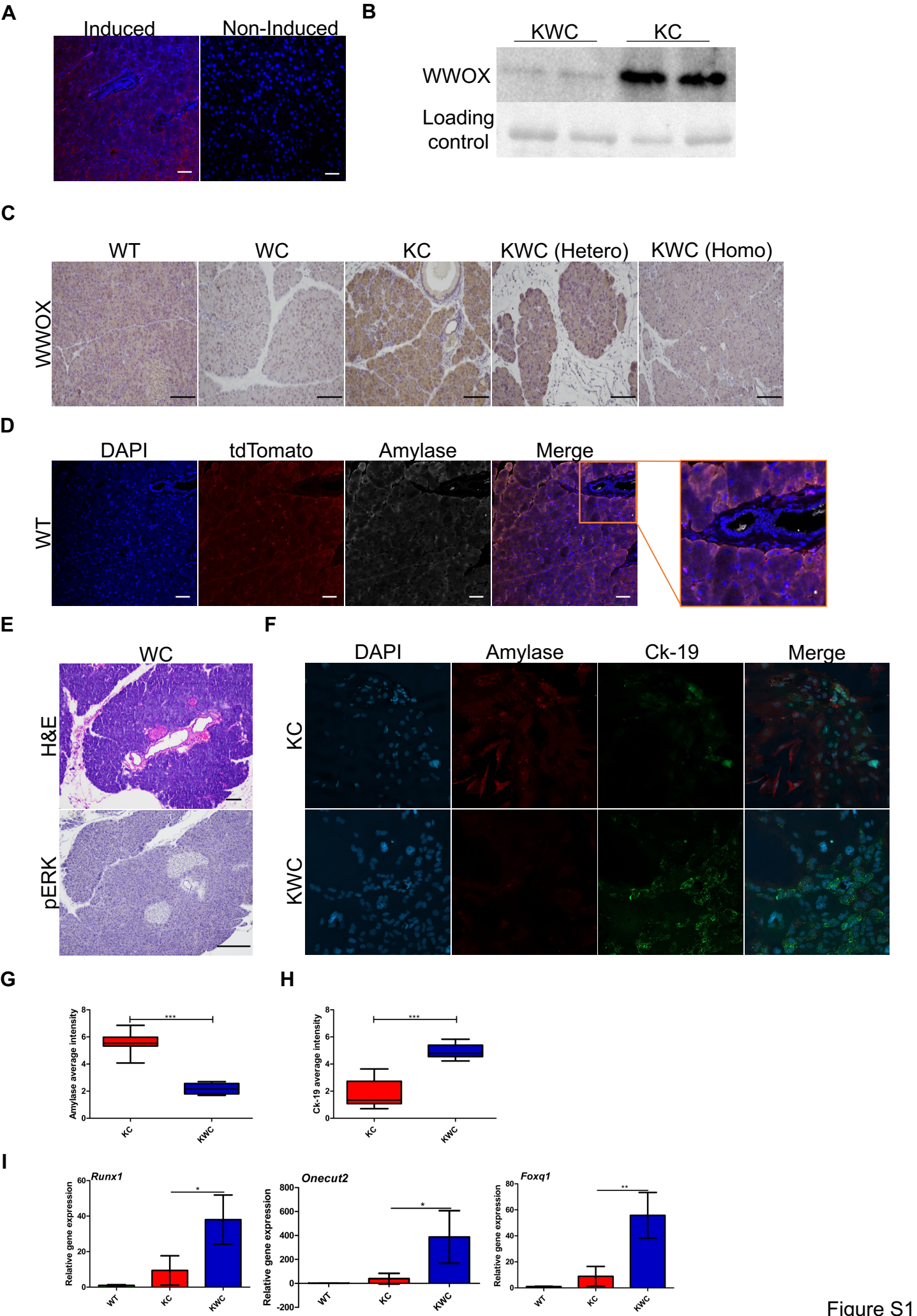

A

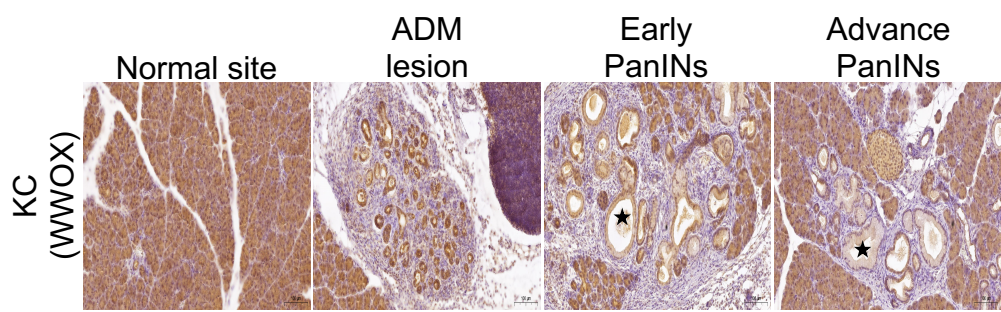

B

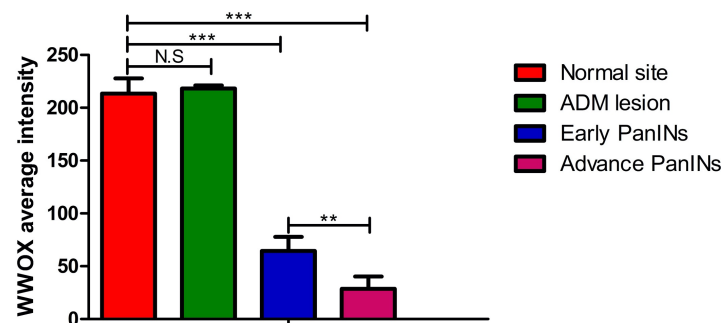

Figure S2

**A**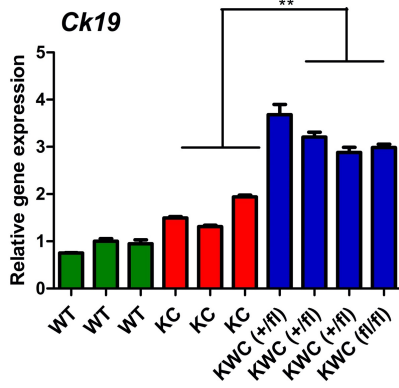**B**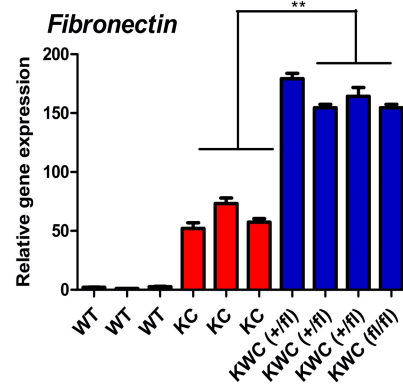**C**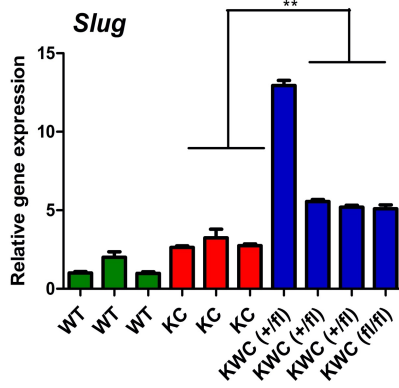**D**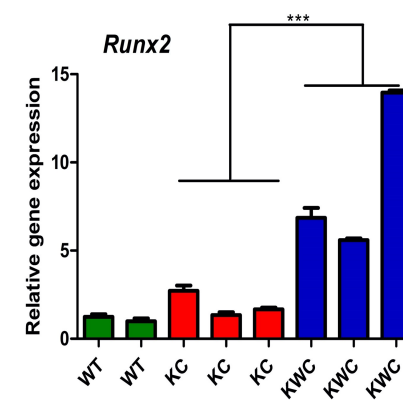**E**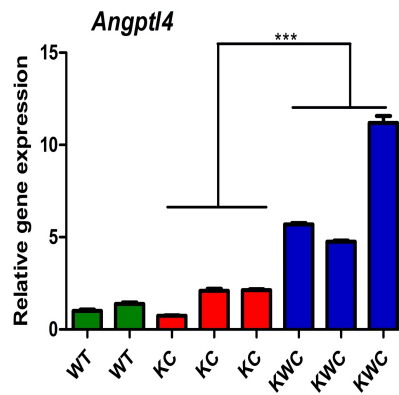

**A**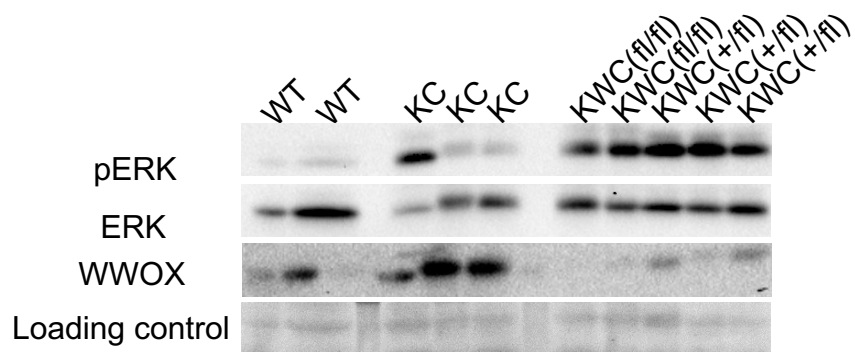**B**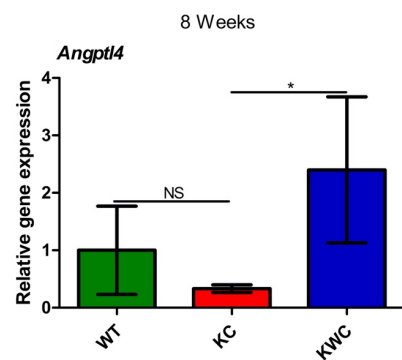**C**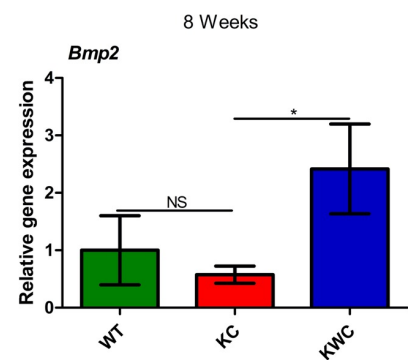**D**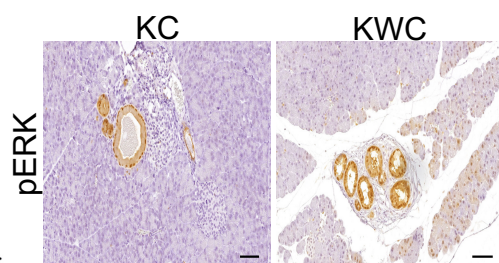**E**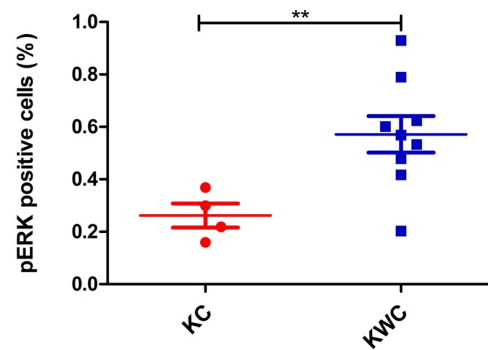

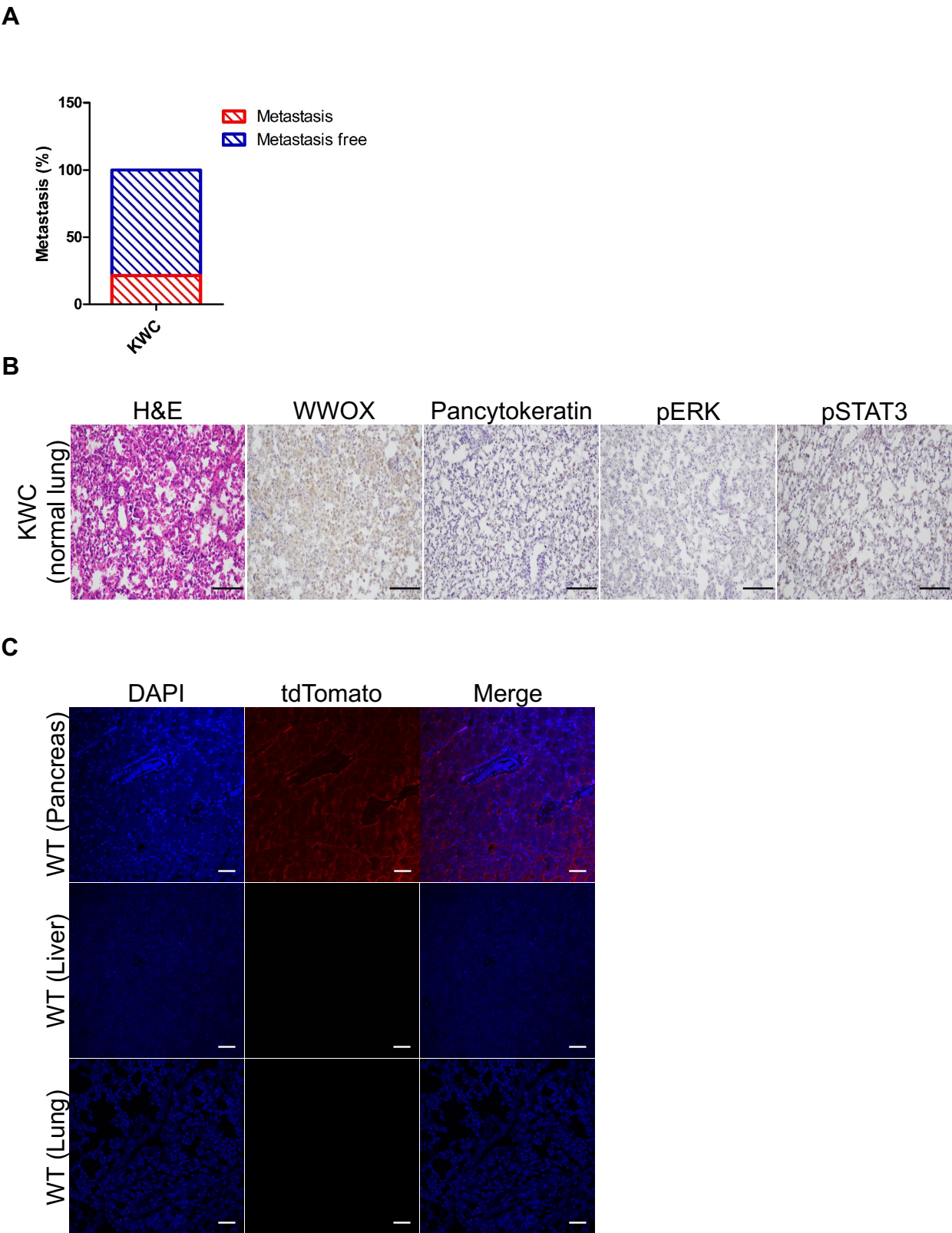

Figure 5S

**A**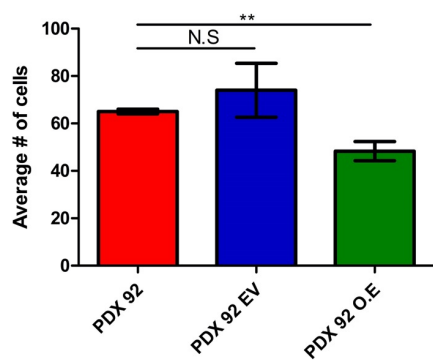**B**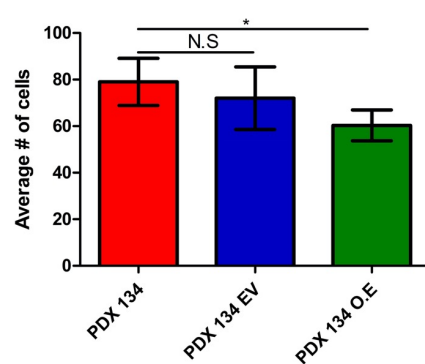**C**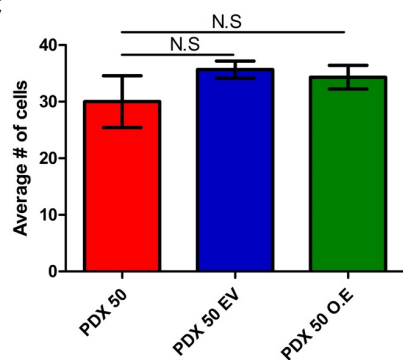**D**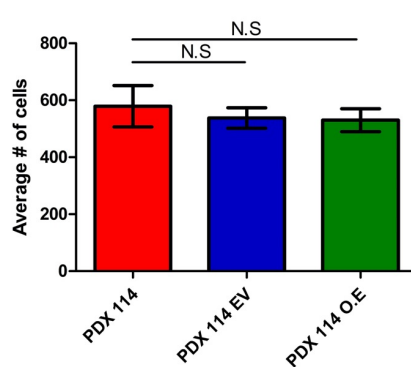**E**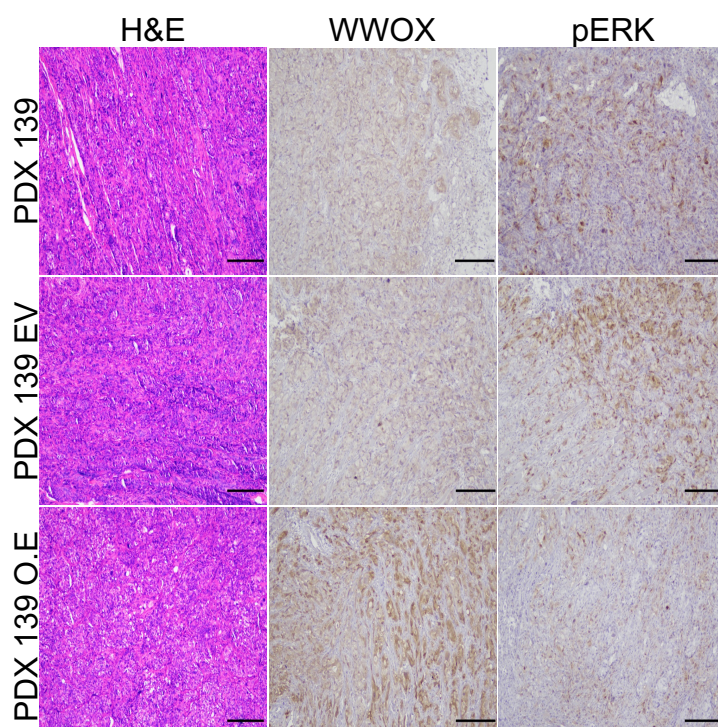

Supplement: Supplementary file 2 — Supplementary FIGURES [file 41419_2022_5519_MOESM2_ESM.pdf]

Fig S1B

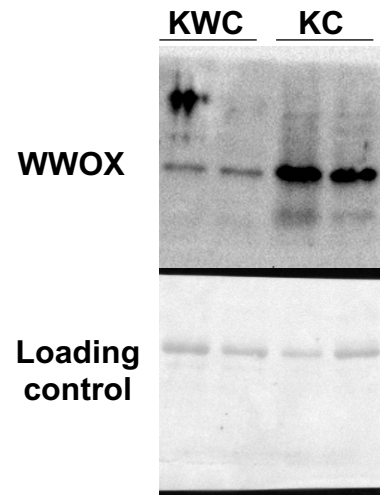

Fig 2B

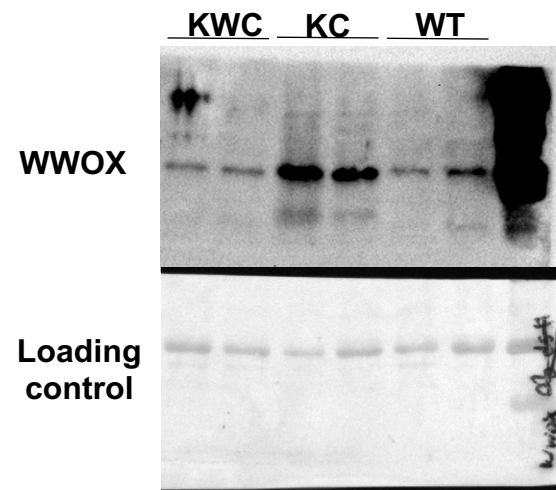

Fig 2D

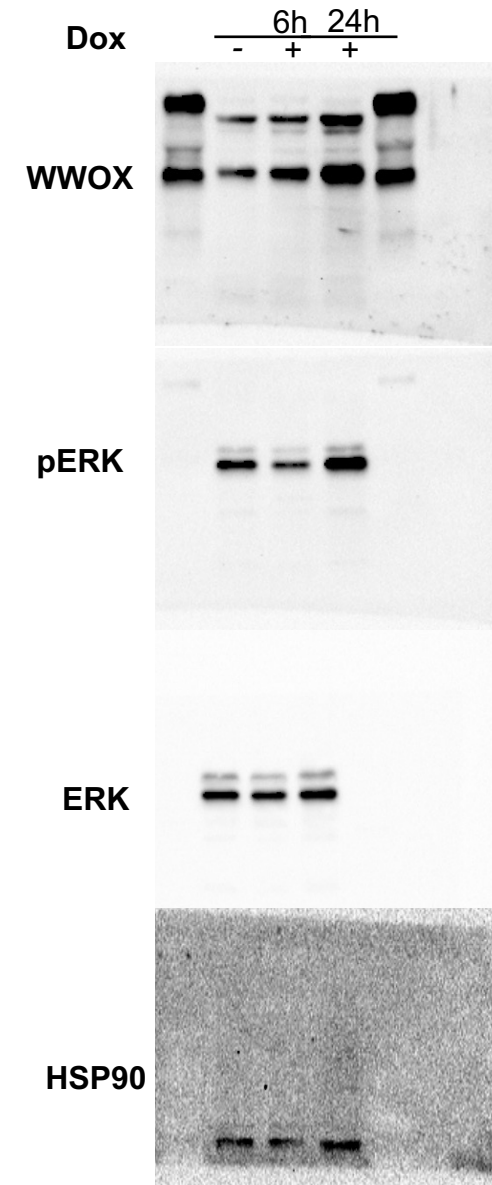

**Fig 4A**

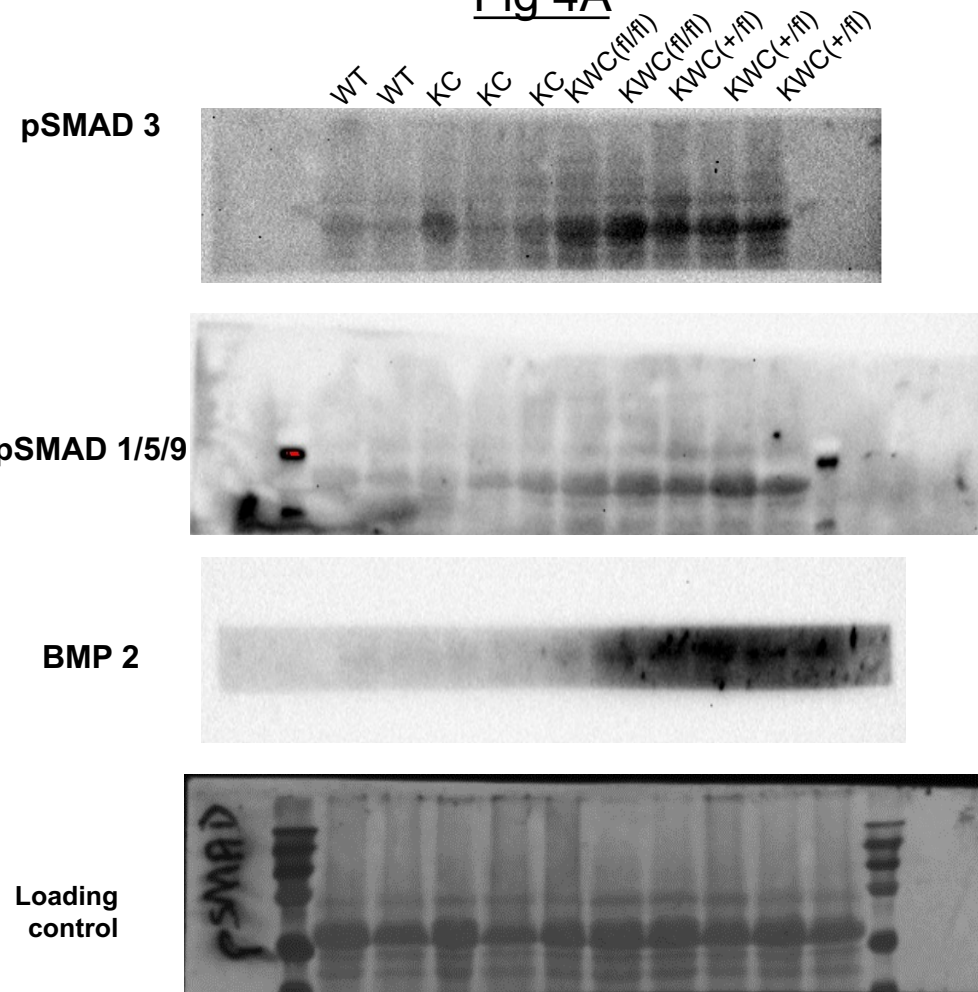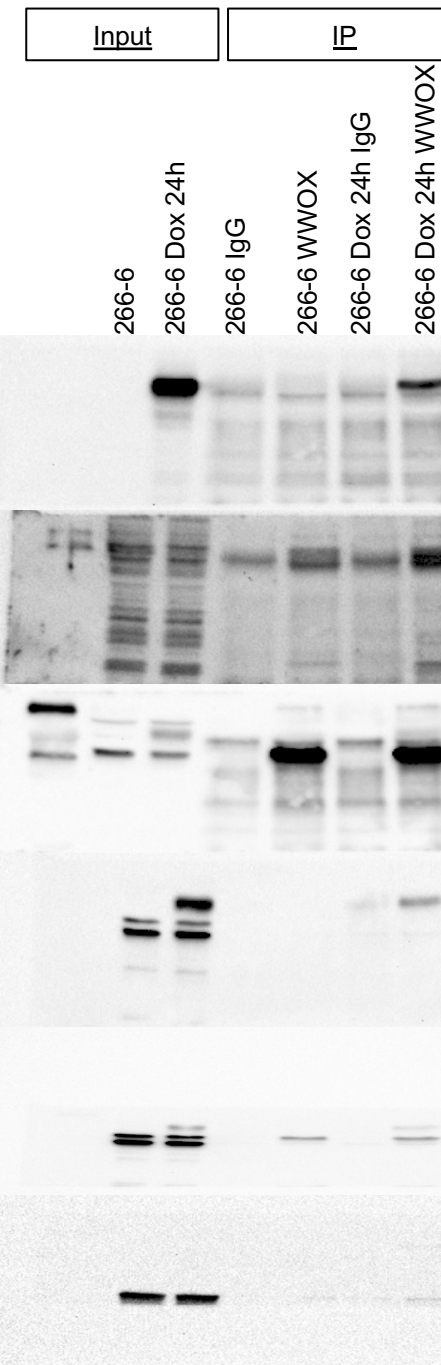

**Fig 4H**

Fig. S4A

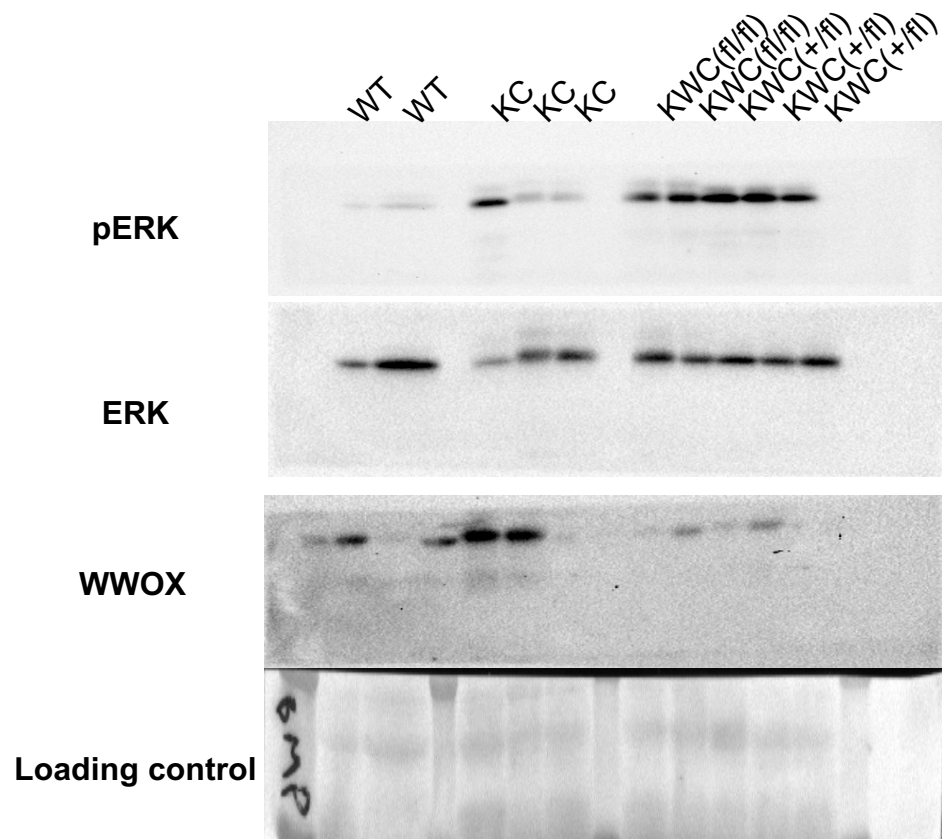

Fig 6B

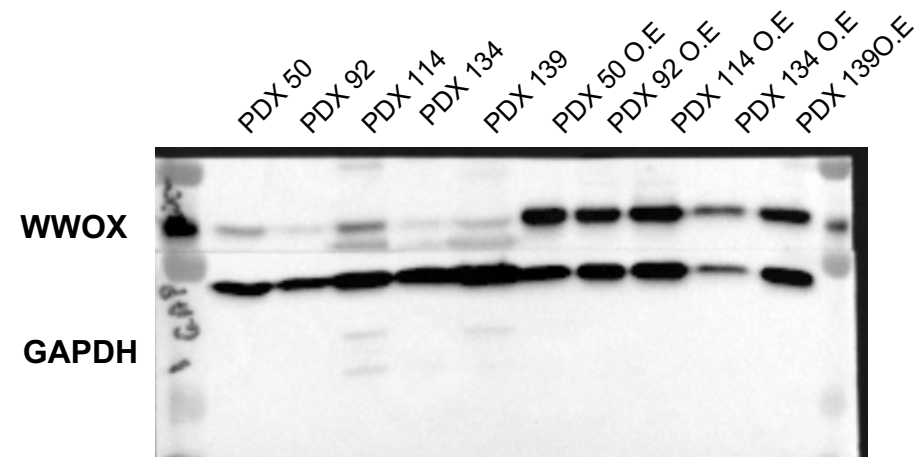

Fig 6G

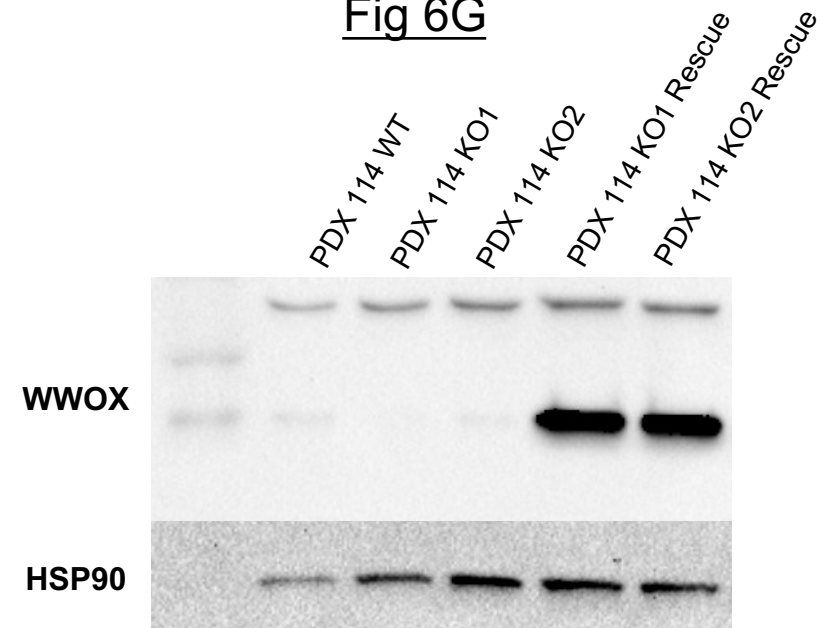

Supplement: Supplementary file 7 — Original Data File [file 41419_2022_5519_MOESM7_ESM.pdf]
