# Supplementary material for: Loss of tumor suppressor WWOX accelerates pancreatic cancer development through promotion of TGFβ/BMP2 signaling
Source: Cell Death Dis. 2022 Dec 27;13(12):1074. doi: 10.1038/s41419-022-05519-9 (PMC9792466; doi:10.1038/s41419-022-05519-9)
Supplement: Supplementary file 3 — Supplementary FIGURES LEGENDS [file 41419_2022_5519_MOESM3_ESM.pdf]

Supplementary Figure Legends

Supplementary Figure 1. Validation and characterisation of KWC mouse model. (A) Immunofluorescence stain of Tomato in tamoxifen induced, and non-induced pancreas. (B) Immunoblotting of WWOX in KWC and KC mice one-month post-tamoxifen injection. (C) Immunochemical stain of WWOX in WT, WC, KC, KWC (hetero), and KWC (homo) respectively two-months post-tamoxifen injection (magnification, ×20). (D) Co-immunofluorescence stain of tomato and amylase in WT mice (magnification, ×40). (E) H&E and pERK staining, respectively, in WC mice (magnification, ×10). (F) Representative amylase and Ck-19 immunofluorescence staining of KC, and KWC mice (magnification, ×10). (G) Quantification of amylase intensity in KC and KWC mice. (H) Quantification of Ck-19 intensity in KC and KWC mice. (I) RNAseq analysis of *Runx1, Onecut2,* and *Foxq1* genes in WT, KC, and KWC mice at two-months post-tamoxifen injection. *P<0.05, **P<0. 001, ***P<0. 0001. Two-tail unpaired t-test. Quantification graphs are presenting the means ± SD.

Supplementary Figure 2. Gradual loss of WWOX expression upon lesions formation. (A) Representative WWOX immunostaining at normal and lesion sites of KC mice eight-months post-tamoxifen injection (magnification, ×40). (B) Quantification of WWOX average intensity at normal sites, ADM sites, early and advanced PanINs. NS>0.05, **P<0. 001, ***P<0. 0001. Two-tail unpaired t-test. Quantification graphs are presenting the means ± SD.

Supplementary Figure 3. RT-PCR of *Ck-19*, *Fibronectin*, *Slug*, *Runx2* and *Angptl4* respectively of WT, KC, and KWC mice one-month post-tamoxifen injection.

Supplementary Figure 4. WWOX deletion induces ERK MAPK signaling through TGFβ and BMP2 signaling pathway. (A) Immunoblotting of WWOX, pERK and ERK of WT (n=2), KC (n=3), KWC *Wwoxf/+* (n=3), and KWC *Wwoxf/f* (n=2) mice at one-month post-tamoxifen injection. Anti-WWOX blot was run separately on a different gel, but same samples. (B) RNAseq analysis of *Angptl4* of WT, KC, and KWC mice two-months post-tamoxifen injection. (C) RNAseq analysis of *Bmp2* of WT, KC, and KWC mice two-months post-tamoxifen injection. (D) Representative IHC staining of pERK of KC, and KWC mice one-month post-tamoxifen injection (magnification, ×40). (E) Quantification of pERK positive cells in KC (n=4), and KWC (n=9) mice. NS>0.05, **P*<0.05, ***P*<0. 001. Two-tail unpaired t-test. Quantification graphs are presenting the means ± SD.

Supplementary Figure 5. Validation of metastasis origin. (A) 3 out of the 16 KWC tumor-bearing mice formed metastasis. (B) Representative WWOX, Pancytokeratin, pERK, pSTAT3, and amylase immunochemical staining respectively in normal lungs of KWC mice (magnification, ×10). (C) Representative immunofluorescence staining of tdTomato of WT pancreas, liver, and lung respectively (magnification, ×40).

Supplementary Figure 6. WWOX restoration suppress growth. (A) Quantification of colony formation of PDX 92, EV, and WWOX O.E, respectively. (B) Quantification of colony formation of PDX 134, EV, and WWOX O.E, respectively. (C) Quantification of colony formation of PDX 50, EV, and WWOX O.E, respectively. (D) Quantification of colony formation of PDX 114, EV, and WWOX O.E, respectively. (E) Representative H&E, WWOX, and pERK stain respectively of PDX 139, PDX 139 EV, and PDX 139 WWOX OE (magnification ×10). NS>0.05, **P*<0.05, ***P*<0. 001. Two-tail unpaired t-test. Quantification graphs are presenting the means ± SD.
